# Sodium/(calcium + potassium) exchanger NCKX4 optimizes KLK4 activity in the enamel matrix microenvironment to regulate ECM modeling

**DOI:** 10.3389/fphys.2023.1116091

**Published:** 2023-02-06

**Authors:** Barry Chan, Ieong Cheng Cheng, Jalali Rozita, Ida Gorshteyn, Yulei Huang, Ida Shaffer, Chih Chang, Wu Li, Jonathan Lytton, Pamela Den Besten, Yan Zhang

**Affiliations:** ^1^ Department of Orofacial Sciences, University of California, San Francisco, CA, San Francisco, United States; ^2^ Guangdong Provincial Key Laboratory of Stomatology, Guanghua School of Stomatology, Sun-Yat-sen University, Guangzhou, China; ^3^ Department of Biochemistry and Molecular Biology, University of Calgary, Calgary, AB, Canada

**Keywords:** enamel biomineralization, ameloblast differentiation, K^+^-dependent Na^+^/Ca^2+^-exchanger, kallikrein-related peptidase 4, extracellular matrix remodeling, proteinase activity

## Abstract

Enamel development is a process in which extracellular matrix models from a soft proteinaceous matrix to the most mineralized tissue in vertebrates. Patients with mutant *NCKX4*, a gene encoding a K^+^-dependent Na^+^/Ca^2+^—exchanger, develop a hypomineralized and hypomature enamel. How NCKX4 regulates enamel protein removal to achieve an almost protein-free enamel is unknown. We characterized the upregulation pattern of *Nckx4* in the progressively differentiating enamel-forming ameloblasts by qPCR, and as well as confirmed NCKX4 protein to primarily localize at the apical surface of wild-type ruffle-ended maturation ameloblasts by immunostaining of the continuously growing mouse incisors, posing the entire developmental trajectory of enamel. In contrast to the normal mature enamel, where ECM proteins are hydrolyzed and removed, we found significant protein retention in the maturation stage of *Nckx4* ^*−/−*^ mouse enamel. The *Nckx4* ^*−/−*^ enamel held less Ca^2+^ and K^+^ but more Na^+^ than the *Nckx4* ^*+/+*^ enamel did, as measured by EDX. The alternating acidic and neutral pH zones at the surface of mineralizing *Nckx4* ^*+/+*^ enamel were replaced by a largely neutral pH matrix in the *Nckx4* ^*−/−*^ enamel. *In situ* zymography revealed a reduced kallikrein-related peptidase 4 (KLK4) activity in the *Nckx4* ^*−/−*^ enamel. We showed that KLK4 took on 90% of proteinase activity in the maturation stage of normal enamel, and that recombinant KLK4 as well as native mouse enamel KLK4 both performed less effectively in a buffer with increased [Na^+^] and pH, conditions found in the *Nckx4* ^*−/−*^ developing enamel. This study, for the first time to our knowledge, provides evidence demonstrating the impaired *in situ* KLK4 activity in *Nckx4* ^*−/−*^ enamel and suggests a novel function of NCKX4 in facilitating KLK4-mediated hydrolysis and removal of ECM proteins, warranting the completion of enamel matrix modeling.

## Introduction

Extracellular matrix (ECM) remodeling is a fundamental development process that contributes to the tissue-specific mechanical and structural properties (Yue, 2014). Dental enamel, a mineralized ECM orchestrated by epithelial-derived ameloblasts, is a tissue designed to protect teeth throughout a lifetime. Enamel undergoes a series of steps to revamp the tissue architecture and ECM composition to produce the hardest substance in our bodies. Once teeth erupt, ameloblasts commit apoptosis, leaving enamel as a non-regenerative tissue (Moradian-Oldak, 2012). Understanding how the enamel matrix is modeled and biomineralized will lay the foundation for the future regeneration of functional ameloblasts and enamel.

Enamel matrix formation goes through two major phases, a proteinaceous matrix phase and a mineralizing phase (Bartlett, 2013). During the first phase, ECM proteins are synthesized and laid out by the polarized secretory ameloblasts (SABs) until the enamel matrix grows to its full thickness between the dentin matrix and a tightly conjunct ameloblast layer. After secretion, enamel matrix proteins are immediately selectively hydrolyzed by MMP20, the predominant matrix metalloproteinase present during the proteinaceous phase of enamel formation (Llano et al., 1997; Begue-Kirn et al., 1998; Caterina et al., 2002; Bartlett et al., 2004; Kim et al., 2005; Bartlett et al., 2011; Yamakoshi et al., 2011).

The major mineralizing phase begins with a brief period of transition ameloblasts. These cells generate a number of proteinases, primarily kallikrein-related peptidase 4 (KLK4), to degrade enamel matrix proteins processed by MMP20 previously (Simmer et al., 2009; Simmer et al., 2011; Yamakoshi et al., 2013; Bartlett and Simmer, 2014). Transition ameloblasts are promptly followed by maturation ameloblasts (MABs), which endocytose and remove KLK4- processed small molecular weight peptides from the ECM, thus making space for mineral deposition and the expansion of crystallized enamel (Nagano et al., 2009). MABs cyclically modulate between cells with a ruffle-ended (with membrane infoldings) and smooth-ended appearance (no membrane infoldings) at their apical border (Warshawsky and Smith, 1974; Josephsen and Fejerskov, 1977). Ruffle-ended and smooth-ended ameloblasts overlay enamel matrix in which pH cycles between 6.2 and 7.2, respectively, correspondingly (Takagi et al., 1998). Ameloblast modulation and underlying enamel matrix pH cycling are prerequisite events ensuring normal enamel formation (Lacruz et al., 2017), but how they are connected remains unclear.

The strength of enamel owes largely to its extraordinary degree of mineralization state. While bone and dentin are composed of about 70% minerals, enamel comprises approximately 96% minerals by weight (Follet et al., 2004). Studies tracing radiolabeled calcium have shown that about 14% of the enamel calcium is fetched by SABs using a trans-ameloblastic route, while MABs are responsible for delivering the remaining 86% of calcium to the enamel (Smith, 1998). Approximately 70% of this calcium is transported by ruffle-ended MABs through a transcellular route, and the rest is delivered through a paracellular route between smooth-ended MABs (Smith, 1998). Clinical genetic analyses have shown that mutations in human genes involved in trans-ameloblastic calcium transport, including *STIM1*, *ORAI1,* and *SLC24A4*, can cause amelogenesis imperfecta (AI), an inherited defect that significantly affects enamel structure and functions (Parry et al., 2013; Seymen et al., 2014; Wang et al., 2014; Herzog et al., 2015; Lacruz and Feske, 2015; Jalloul et al., 2016; Prasad et al., 2016). STIM1 and ORAI1 are primarily responsible for calcium entry into ameloblasts from the serosal compartment at their basal end (Furukawa et al., 2017; Nurbaeva et al., 2017; Smith et al., 2017). *SLC24A4* (solute carrier family 24, member 4) is a gene symbol approved by HUGO. Six mutations in the human *SLC24A4* gene have been linked with AI, accounting for about 1% of total AI cases (Jalloul et al., 2016).

SLC24A4 is also known as NCKX4, a member of the potassium-dependent sodium/calcium exchanger family. Members of this protein family possess eleven transmembrane helices, including two pairs referred to as alpha-1 and alpha-2 repeats, which form the ion-binding and transport pockets (Altimimi and Schnetkamp, 2007; Lytton, 2007; Parry et al., 2013). NCKX proteins function to exchange 4Na^+^ inward against 1Ca^2+^ plus 1K^+^ outward (Schnetkamp, 1989). NCKX4 is expressed in various tissues, including the brain, lung, thymus, and the olfactory and visual sensory neurons (Li et al., 2002). Therefore, in each of these tissues, NCKX4 may play specific roles related to microenvironment Ca^2+^ homeostasis (Altimimi and Schnetkamp, 2007). For instance, NCKX4-mediated extrusion of Ca^2+^ from the cilia of olfactory receptor cells shapes the olfactory response and mediates sensory adaptation (Stephan et al., 2011). As NCKX4 is significantly upregulated in mouse maturation ameloblasts (Hu et al., 2012; Wang et al., 2014; Bronckers et al., 2015), it is a good candidate for regulating the massive calcium extrusion required for the mineralization of the enamel ECM (Hu et al., 2012; Bronckers et al., 2015; Lacruz, 2017). However, the molecular mechanisms underlying the formation of hypomaturation type of amelogenesis imperfecta in the patients carrying *SLC24A4 (NCKX4)* mutation are unclear.

We hypothesize that, in addition to functioning as a calcium/sodium exchanger, NCKX4 also participates in ECM protein hydrolysis and removal during enamel development to achieve the highest degree of biomineralization. In this study, we utilized the continuously growing mouse incisors that allow us to investigate the complete trajectory of enamel ECM protein hydrolysis and removal in adult animals. An NCKX4 null mouse model (*Nckx4* ^*−/−*^) with deletion *Nckx4* ^*−/−*^ of *Nckx4* exon 6 and 7 (Li and Lytton, 2014) provided us the opportunity to investigate the specific functions of NCKX4 in enamel matrix modeling during amelogenesis. We found that the activity of KLK4 was optimized in the slightly acidic and low sodium microenvironment, which can be found in the developing enamel matrix overlaid by wild-type ruffle-ended maturation ameloblasts. In the *Nckx4* ^*−/−*^ mouse enamel matrix, which had an increased amount of Na^+^ and disrupted pH cycling, KLK4 activity was significantly compromised compared to that in *Nckx4* ^*−/−*^enamel. *In vitro* analyses validated that the activity of recombinant and mouse enamel native KLK4 was suppressed by the elevated [Na^+^] and pH. Our analyses demonstrate a novel function of NCKX4 in creating an optimized microenvironment for KLK4 to effectively hydrolyze proteins of the enamel matrix and promote its subsequent biomineralization and describe a previously uncharted molecular mechanism responsible for the hypomaturation type of enamel defect in *SLC24A4* (*NCKX4*) gene mutants.

## Results

### NCKX4, significantly upregulated in the early maturation ameloblasts, cyclically redistributes between the ruffle-ended and smooth-ended maturation ameloblasts

**FIGURE 1:**
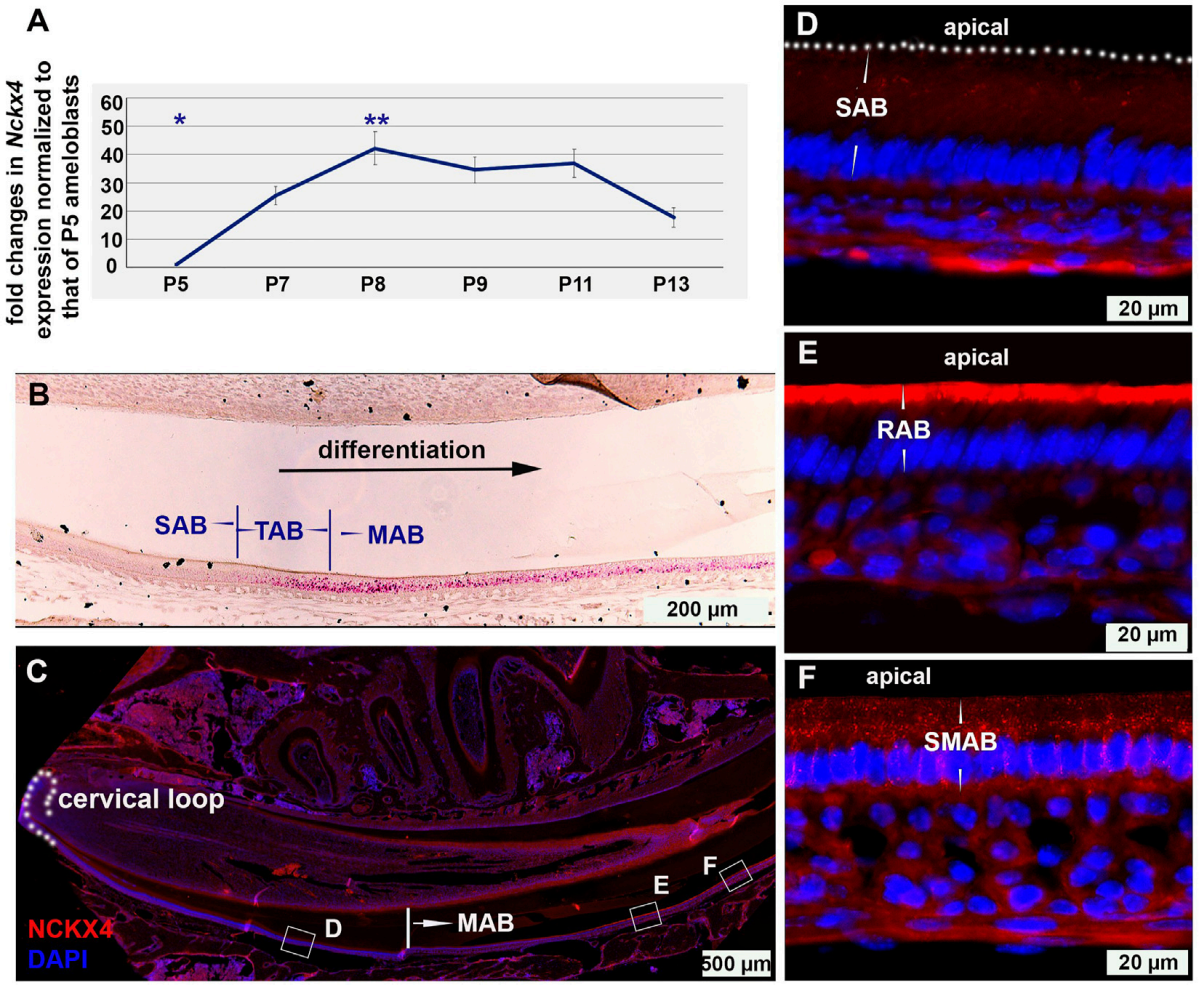
The temporal and spatial expression of NCKX4 in ameloblast lineage. **(A)** Semi-quantitative PCR analysis on the expression of *Nckx4* in the dissected ameloblasts at secretory (P5), transition (P7), early maturation (P8), mid-maturation (P9-P11), and late maturation (P13) stage of development. The average qPCR cycle threshold (Ct) of endogenous control *Gapdh* of P5 SAB is 20.4, and that of *Nckx4* of P5 SAB is 28.5. The fold changes in the expression levels of *Nckx4* in those more differentiated ameloblasts were normalized to the levels of secretory ameloblasts (P5), *n* = 4 per time point. Each n included the cells from 5 mice. The significance of differences was determined by one-way ANOVA analysis using SPSS Statistics package 19 followed by Tukey Post Hoc Tests. *p < 0.05 compared to P7, P8, P9, P11, and P13. **p < 0.05 compared to P5, P7, and P13. **(B)** *In situ* hybridization was used to assess the expression and distribution of *Nckx4* mRNA in secretory ameloblasts (SAB), transition ameloblasts (TAB), and maturation ameloblasts (MAB) on the sagittal sections of wild-type mouse incisors (*n* = 3). **(C)** Immunostaining of NCKX4 along the trajectory of ameloblast differentiation, from dental epithelial stem cells residing in the cervical loop, secretory, transition, to ruffle-ended and smooth-ended maturation ameloblasts, fully coexisting in the continuously growing mouse incisors (*n* = 3). **(D–F)** Magnified images taken from the regions boxed in panel C. The NCKX4 signal was relatively weak in secretory ameloblasts (SAB; panel D), primarily concentrated at the apical surface of ruffle-ended maturation ameloblasts (RAB; panel E), and sparsely distributed in the central cytosolic region of the smooth-ended maturation ameloblasts (SMAB; panel F).

To correlate the expression pattern of *Nckx4* with ameloblast functions, ameloblasts of enamel organs at the various developmental stages were collected for semi-quantitative PCR. We found that as compared to secretory ameloblasts (P5), *Nckx4* expression started to increase in the post-secretory ameloblasts (P7) and reached a peak at the early maturation stage (P8), followed by a reduction at the late maturation stage (see Figure 1A). Overall, the expression levels of *Nckx4* in maturation ameloblasts were significantly greater than that in secretory ameloblasts. The pattern of *Nckx4* expression in the maturation stage ameloblasts, in particular in the early maturation ameloblasts, was confirmed by *in situ* RNA hybridization (RNAscope) (see Figure 1B).

Immunostaining on the full spectrum of ameloblast lineage on incisal sagittal sections showed that the NCKX4 signal apparently increased in the transition ameloblasts compared to secretory ameloblasts (Figure 1D), peaked in the early maturation ameloblast (MAB), and slightly reduced at the end of maturation stage (Figure 1C). In maturation stage ameloblasts, NCKX4 was primarily concentrated along the apical end of ruffle-ended ameloblasts (RAB) (see Figure 1E), but scattered in the cytosol of smooth-ended ameloblasts (SMAB) with reduced intensity (see Figure 1F). A recapture of the temporal and spatial expression pattern of NCKX4 aids us to understand its stage- and phase-specific functions in the enamel maturation.

### NCKX4 is required for enamel biomineralization and enamel rod formation

**FIGURE 2:**
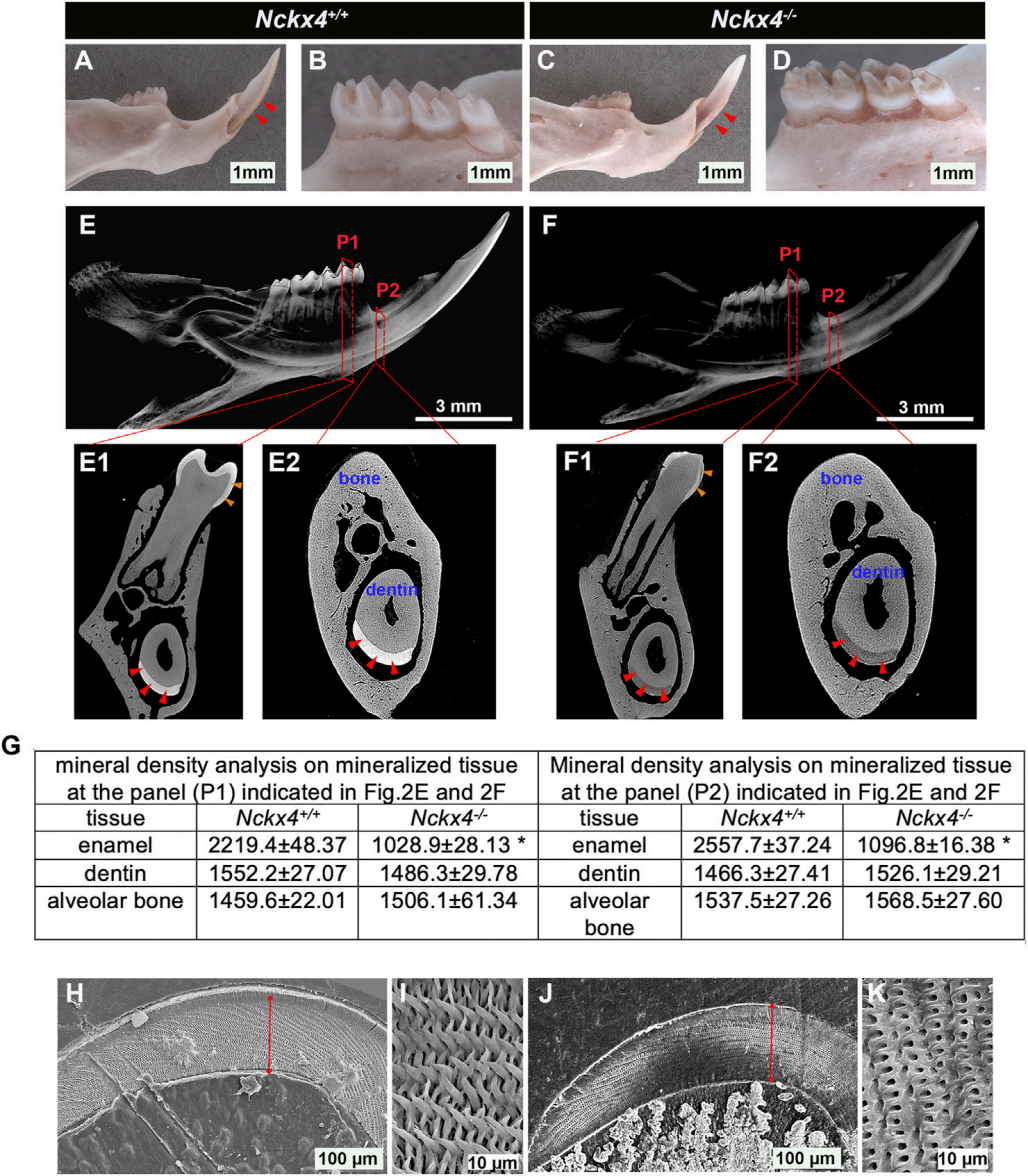
Characterization of enamel appearance, mineral density, and microstructure. **(A)** A representative image of the P7W *Nckx4* ^*+/+*^ mouse hemimandibular incisor with a sharp tip and pigmented outer enamel (indicated by red triangles). **(B)** A representative image of the *Nckx4* ^*+/+*^ mouse hemimandibular molars covered with well-formed cusps and translucent outermost enamel. **(C)** A representative image of P7W *Nckx4* ^*−/−*^ hemimandibular incisor on which white enamel was chipped off, only a small portion of enamel retained adjacent to the gingival margin region (indicated by red triangles). **(D)** A representative image of *Nckx4* ^*−/−*^ mouse hemimandibular molars on which the cusps were worn out and the outer enamel was heavily stained in brown. **(E)** A representative reconstituted 3D microCT image of the P7W *Nckx4* ^*+/+*^ mouse hemimandibles showed the radiopaque enamel layer on both molars and incisor. **(F)** A representative reconstituted 3D microCT image of the P7W *Nckx4* ^*−/−*^ mouse hemimandibles showed no well-contrasted radiopaque layer on both molars and incisor. **(E1)** A cross-section of the second buccal cusps of the *Nckx4* ^*+/+*^ first molar, designated as P1 in panel E, showed radiopaque enamel layers on the first molar (indicated by orange triangles) and on the incisor (indicated by red triangles). **(E2)** A cross-section of the onset of the mesial angle of the first molar, designated as P2 in panel E, showed the enamel layer on the incisor (indicated by red triangles). **(F1)** A cross-section at P1 in panel F shows less radiopaque enamel layers on the first molar (indicated by orange triangles) and on the incisor (indicated by red triangles). **(F2)** A cross-section at P2 in panel F shows the enamel layer on the incisor (indicated by red triangles). **(G)** The mineral density measured by EDX in the P7W *Nckx4* ^*−/−*^ mouse enamel was reduced to 53% of that in the P7W *Nckx4* ^*+/+*^ enamel in P1, and 57% in P2, *p < 0.05, *n* = 4, determined by unpaired two-tailed Student’s *t*-test. **(H)** Scanning electron microscopy (SEM) analysis of the P7W *Nckx4* ^*+/+*^ developing enamel on which the full thickness enamel layer is marked by a red line. **(I)** A representative magnified SEM image of the P7W *Nckx4* ^*+/+*^ enamel showed that the well-aligned enamel crystals were bundled together as individual enamel rods, arranged in a decussating pattern. **(J)** The thickness of the *Nckx4* ^*−/−*^ developing enamel (marked by a red line) was comparable to that of the *Nckx4* ^*+/+*^ enamel. **(K)** A representative magnified SEM image of the *Nckx4* ^*−/−*^ enamel showed the decussating arrangement of microstructures and increased enamel space between the empty holes. These holes represent the etched and removed minerals along the rod axis.

As compared to the smooth and translucent enamel of *Nckx4* ^*+/+*^ incisor (see Figure 2A) and molars (see Figure 2B), the enamel layers on both *Nckx4* ^*−/−*^ incisor (see Figure 2C) and molars (see Figure 2D) were worn, chipped away and heavily discolored. These appearances are also found on the teeth of patients diagnosed with amelogenesis imperfecta.

MicroCT scanning showed that the enamel layer in both sagittal (see Figure 2E) and cross sections (see Figures 2E1, E2) of *Nckx4* ^*+/+*^ hemimandibles was radiopaque (indicated by red triangles in Figures 2E1, E2 for incisal enamel, orange triangles for molar enamel), and was well contrasted to the dentin and surrounding alveolar bone. Both enamel on incisors (indicated by red triangles in Figures 2F1, F2) and on molar (indicated by orange triangles in Figure 2F1) of *Nckx4* ^*−/−*^ mice had a significantly reduced radiopacity. The mineral density in the *Nckx4* ^*−/−*^ mouse enamel was reduced to 53% of that in the *Nckx4* ^*+/+*^ enamel in P1, and 57% in P2, *n* = 4, *p < 0.05, determined by unpaired two-tailed Student’s *t*-test (see Figure 2G).

Cross sections of mouse hemimandibles at the planes indicated by P2 in Figures 2E, F showed that there was no significant alteration in the thickness of *Nckx4* ^*−/−*^ late maturation stage enamel as compared to that in *Nckx4* ^*+/+*^ matrix (see Figures 2H, J). SEM analysis revealed a normal decussating pattern of enamel prisms in both *Nckx4* ^*+/+*^ and *Nckx4* ^*−/−*^ developing enamel. However, compared to the well-developed independent enamel rod in *Nckx4* ^*+/+*^ matrix (see Figure 2I), no individualized enamel rod was formed in *Nckx4* ^*−/−*^ enamel matrix (See Figure 2K). Therefore, NCKX4 is critical for the formation of enamel ECM with normal composition and microstructure.

### Loss-of-function of NCKX4 results in alteration of enamel matrix protein removal and ameloblast morphology

**FIGURE 3:**
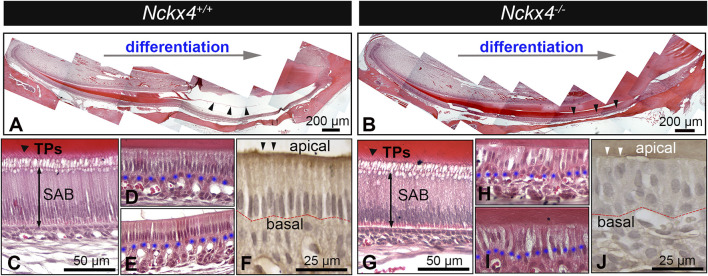
Histological analysis on ameloblasts and enamel extracellular matrix (ECM). **(A)** A representative H&E-stained sagittal section of the P7W *Nckx4* ^*+/+*^ mouse incisors showed that throughout the differentiation process, the enamel organic matrix gradually increased, reached its full thickness, then progressively diminished as indicated by the black triangles. **(B)** A representative sagittal section of the P7W *Nckx4* ^*−/−*^ mouse incisors showed that the enamel organic matrix gradually increased, reached its full thickness, then remained throughout the maturation stage, as indicated by the black triangles. **(C)** A magnified image showed the elongated secretory ameloblasts (SAB) and “picket-fence” shaped Tomes’ processes (TPs) at the apical end of *Nckx4* ^*+/+*^ SAB. **(D)** A magnified image showed the reduced *Nck×4* ^*+/+*^ ruffled-ended ameloblasts and the foaming vacuolization in the apical cytoplasm. **(E)** A magnified image of the reduced *Nck×4* ^*+/+*^ smooth-ended ameloblasts. **(F)** Immunostaining showed the epithelial cell polarity biomarker PKC alpha (indicated by black triangles) present at the apical of *Nckx4* ^*+/+*^ maturation ameloblasts, which remained in a single-cell layer and maintained a well-defined boundary with papillary layer cells at their basal surface (outlined by red dotted line). **(G)** A magnified image of *Nckx4* ^*−/−*^ secretory ameloblasts (SAB) showed that their morphology resembled what is seen in panel C. **(H)** When advancing to the maturation stage, *Nckx4* ^*−/−*^ ameloblasts lost their single-cell layer arrangement, and the boundary with papillary layer cells was unclear. **(I)** In the late maturation stage, the enamel organic matrix was layer still retained, which are supposed to diminish in the normal development seen in panel E. **(J)** PKC alpha was not immunolocalized at the apical of *Nckx4* ^*−/−*^ maturation ameloblasts (indicated by white triangles). These ameloblasts were rearranged into a multiple-cell layer. The blue dotted lines in panel D,E,H, and I outline the boundary between maturation ameloblasts and the papillary layer.

H&E stained sagittal sections of *Nckx4* ^*+/+*^ hemimandibles showed a pink colored enamel matrix that gradually grew to its full thickness, then disappeared at the late development stage. The remaining EDTA demineralized enamel space is indicated by black triangles (see Figure 3A). H&E stained *Nckx4* ^*−/−*^ sections showed a similar secretory stage enamel matrix, but when advancing to its late maturation stage, the enamel matrix in *Nckx4* ^*−/−*^ mice remained present (indicated by the black triangles in Figure 3B).

*Nckx4* ^*+/+*^ secretory ameloblasts (SAB) were elongated and polarized with the characteristic Tomes’ processes (TPs) developed at their apical surface (see Figure 3C), which are responsible for the EMP secretion and decussating pattern of enamel prisms (Zhang et al., 2019). There were no obvious morphological differences between *Nckx4* ^*+/+*^ and *Nckx4* ^*−/−*^ secretory ameloblasts (SAB) (see Figure 3G). When *Nckx4* ^*+/+*^ ameloblasts differentiated to maturation stage, as expected, their height reduced to the half of SAB, and there was a well-defined boundary from papillary layer cells at their basal surface (see Figures 3D, E). Ruffle-ended ameloblasts were identifiable as a classic appearance of a foamy vacuolization was present in their apical cytoplasm (Josephsen and Fejerskov, 1977) (see Figure 3D), while smooth-ended ameloblasts had increased intercellular spaces (see Figure 3E). However, no distinguishable ruffle-ended and smooth-ended ameloblasts were found in the *Nckx4* ^*−/−*^ maturation stage development. In addition, MAB lost their single-cell layer arrangement, and boundary from papillary layer cells was unclear.

The enamel matrix layer was retained throughout *Nckx4* ^*−/−*^ maturation stage (see Figures 3H, I). Epithelial cell polarity biomarker protein kinase C (PKC) alpha was immunolocalized at the apical of *Nckx4* ^*+/+*^ MAB (see Figure 3F), but not in the *Nckx4* ^*−/−*^ MAB (see Figure 3J).

### Amelogenin proteins are retained in the *Nckx4* ^*−/−*^ mouse enamel matrix

**FIGURE 4:**
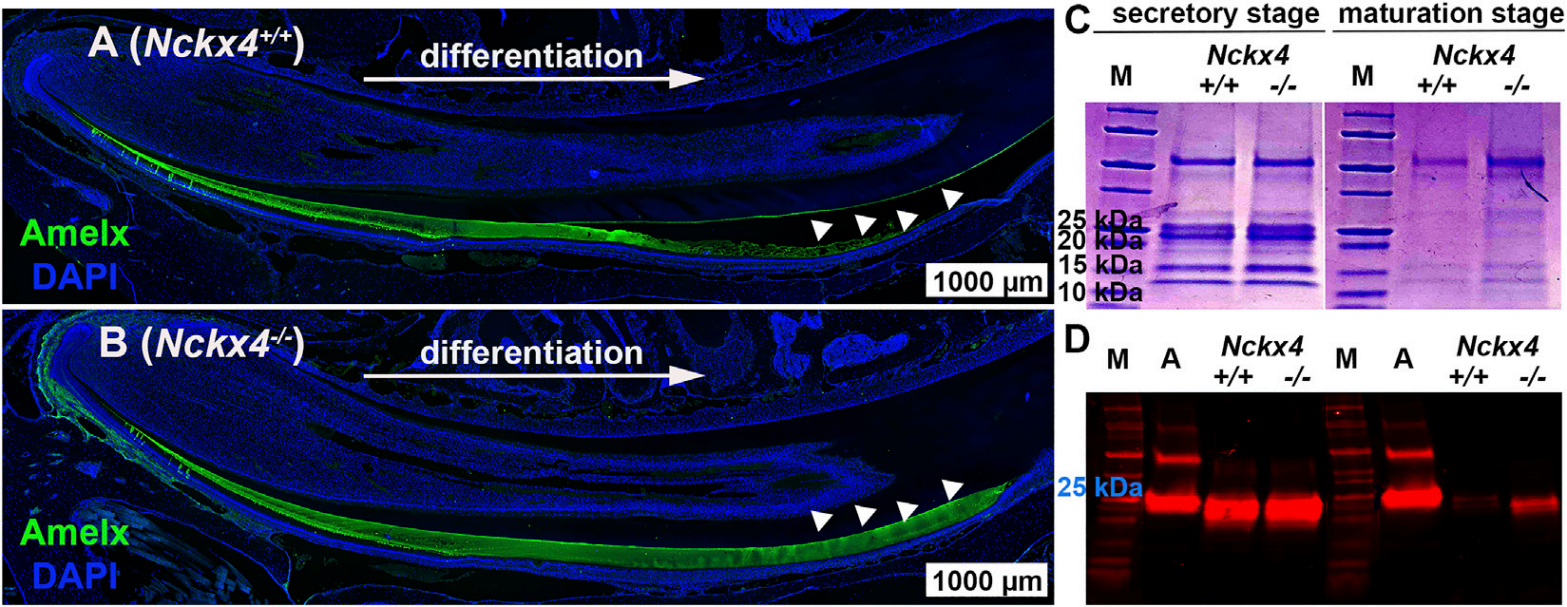
Characterization of ECM organic components in the mouse incisor enamel. **(A)** Immunostaining analysis of sagittal sections of the P7W *Nckx4* ^*+/+*^ mouse incisors showed that the amelogenin immunoreactive signal (green) gradually increased as ameloblasts continuously differentiated, reached maximal thickness, grew thinner, and at last disappeared (indicated by the white triangles). This pattern resembled the dynamic development of enamel ECM presented in Fig.3A since most of ECM proteins there were amelogenins. **(B)** In the P7W *Nckx4* ^*−/−*^ mouse incisor, amelogenin-containing matrix gradually thickened as development proceeded but remained the same thickness all the way through (indicated by white triangles). **(C)** SDS-PAGE analysis of ECM proteins collected from the P7W *Nckx4* ^*+/+*^ and *Nckx4* ^*−/−*^ mouse incisor enamel showed no detectable difference in either protein bands or band density at the secretory stage of development. However, at the maturation stage of development of the *Nckx4* ^*−/−*^ enamel, apparently more proteins with molecular weight less than 25 kDa were detected than that in *Nckx4* ^*+/+*^ controls. **(D)** Western blot analysis showed that amelogenin with a mass of 25 kDa was similarly present in the secretory stage of *Nckx4* ^*+/+*^ and *Nckx4* ^*−/−*^ enamel matrix, but a greater amount of amelogenin 20 kDa was retained in the *Nckx4* ^*−/−*^ maturation phase of enamel compared to *Nckx4* ^*+/+*^. Recombinant full-length amelogenin protein (amelogenin 25 kDa) was used as a positive control.

Immunostaining on *Nckx4* ^*+/+*^ enamel matrix showed a reduced amelogenin immunoreactivity that finally disappeared as protein was removed and replaced by mineral in the mature enamel matrix (see white triangles in Figure 4A). In contrast, in *Nckx4* ^*−/−*^ mouse enamel, amelogenin immunostaining signal continued through the secretory stage, and remained even at the end of maturation stage (see Figure 4B).

SDS-PAGE showed no difference in the secretory stage enamel matrix proteins of *Nckx4* ^*+/+*^ mice as compared to *Nckx4* ^*−/−*^ mice (see Figure 4C), presenting mostly full-length amelogenin with a mass of 25 kDa, and amelogenin without the C-terminus (removed by MMP20) with a mass of 20 kDa (so called amelogenin 20). However, a considerable amount of matrix proteins remained in the maturation stage of the *Nckx4* ^*−/−*^ enamel matrix. Western blot analysis further showed that amelogenin variants in the secretory stage of *Nckx4* ^*−/−*^ enamel were similar to those in the *Nckx4* ^*+/+*^ enamel, but more amelogenin 20 was detected in the maturation stage of *Nckx4* ^*−/−*^ enamel as compared to controls (see Figure 4D). The density analyses of the SDS-PAGE and WB data confirmed significantly more enamel matrix proteins, particularly amelogenins, retained in the *Nckx4* ^*−/−*^ maturation stage of enamel matrix compared to *Nckx4* ^*+/+*^ enamel (see Supplementary Figure S1). These results suggest that NCKX4 is necessary for processing and removing protein components as the enamel ECM models from a protein matrix to a mineralized matrix, a novel function of NCKX4 that has not been previously described.

### Loss-of-function of NCKX4 increases KLK4 expression while decreasing the activity of KLK4 in enamel matrix.

During the maturation phase, KLK4 is the primary enzyme to hydrolyze amelogenin and other EMPs, allowing proteins to be removed and replaced by mineral (Smith, 1998; Ryu et al., 2002). The retention of enamel matrix proteins, in particular amelogenins in *Nckx4* ^*−/−*^ maturation enamel, led us to investigate the effect of NCKX4 deletion on the KLK4 expression and activity.

**FIGURE 5:**
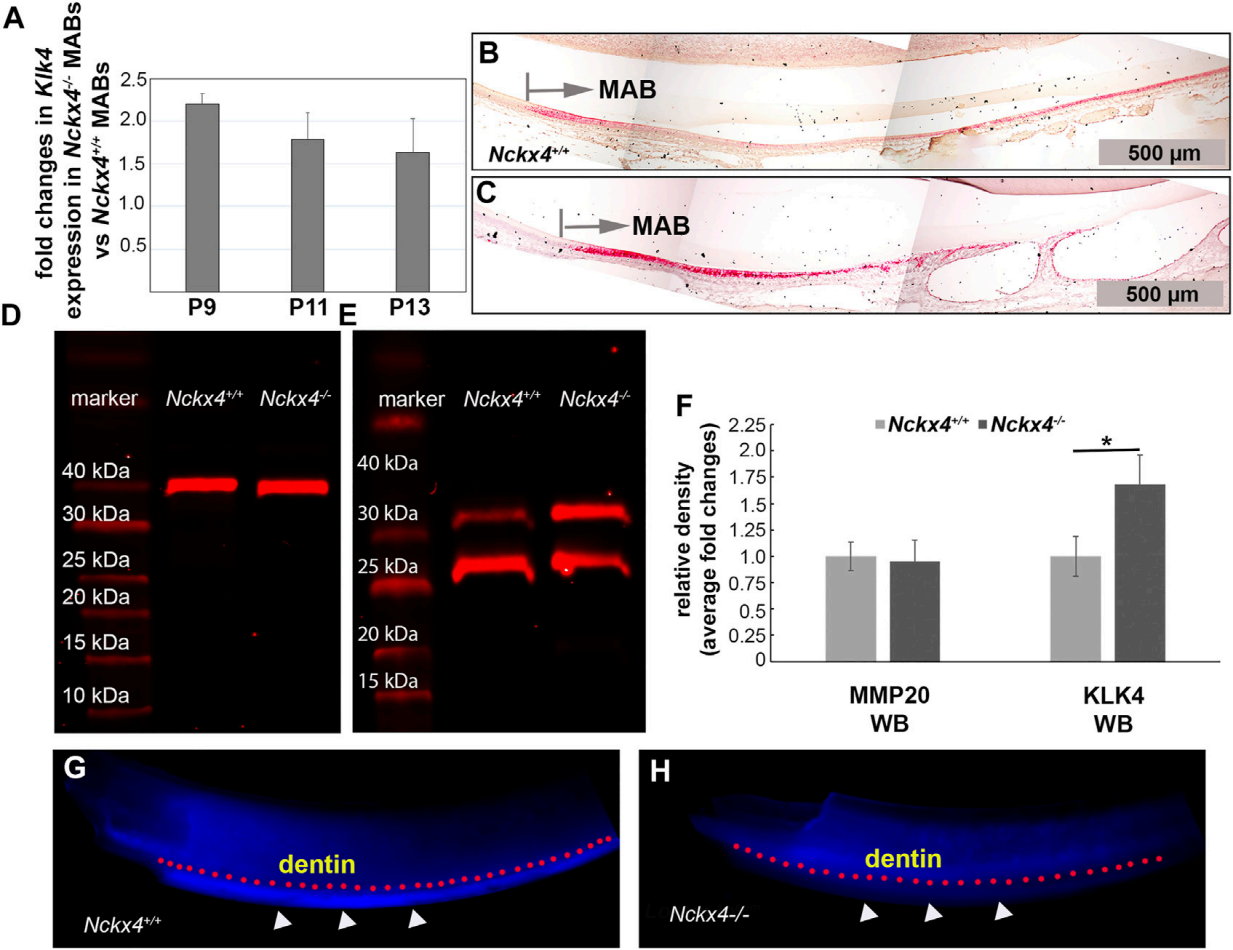
Characterization of *Klk4* mRNA expression in ameloblasts and KLK4 enzyme activity in the enamel matrix. **(A)** Semi-quantitative PCR analysis showed fold increase of *Klk4* mRNA in the *Nckx4* ^*−/−*^ ameloblasts compared to corresponding *Nckx4* ^*+/+*^ ameloblasts at P9, P11, and P13 days of development (*n* = 4, each n containing cells pooled from 5 mice). **(B)** *In situ* hybridization analysis of the P7W *Nckx4* ^*+/+*^ mouse incisors showed increased *Klk4* mRNA signals (in red) in the maturation ameloblasts (MAB), but the magnitude of increase progressively reduced. **(C)** Positive *Klk4* mRNA signals were enhanced in the P7W *Nckx4* ^*−/−*^ mouse MABs as compared to the same developmental stage of *Nckx4* ^*+/+*^ MABs in panel **(B) (D)** Western blot analysis showed that the abundance of MMP20 in the *Nckx4* ^*−/−*^ secretory stage enamel matrix was comparable to that in the *Nckx4* ^*+/+*^ enamel matrix. **(E)** Western blot analysis showed more KLK4 proteins (intact KLK4 and its partially hydrolyzed KLK4 fragment) present in the maturation stage of *Nckx4* ^*−/−*^ enamel compared to that in the *Nckx4* ^*+/+*^ enamel matrix. **(F)** The protein band density analyses of the western blot results in panels D and E quantified that NCKX4 deletion resulted in a greater amount of KLK4 in the *Nckx4* ^*−/−*^ enamel than in the *Nckx4* ^*+/+*^ enamel (*n* = 3, each n containing the enamel matrix from three mice, *p < 0.05, determined by unpaired two-tailed Student’s *t*-test). **(G)** *In situ* KLK4 enzymatic analysis demonstrated that the *Nckx4* ^*+/+*^ maturation stage of enamel matrix was able to hydrolyze Arg-AMC bond in the quenched peptide BOC-Val-Pro-Arg-AMC to release fluorogenic AMC, indicated by the white triangles. A pink dotted line was drawn to outline the boundary between dentin and enamel. **(H)** *In situ* KLK4 analysis showed that much less fluorescence signal was released after the *Nckx4* ^*−/−*^ maturation stage of enamel matrix was incubated with BOC-Val-Pro-Arg-AMC peptide.

Semi-quantitative PCR analyses on mRNA extracted from enamel organ at the transition (P9), early maturation (P11) and middle maturation stage (P13) showed that an increase in *Nckx4* ^*−/−*^ expression compared to *Nckx4* ^*+/+*^ ameloblasts at all three development stages. There was an average of 2.3-fold increase in P9 cells, 1.7-fold in P11 cells, and 1.28-fold in P13 cells (see Figure 5A). The increase of *Klk4* mRNA in *Nckx4* ^*−/−*^ maturation ameloblasts was confirmed by *in situ* hybridization (see Figures 5B, C).

Western blot showed no effects of *Nckx4* deletion on the protein abundance of MMP20 in the secretory stage enamel matrix (see Figure 5D). However, consistent with increased *Klk4* mRNA expression levels, there was relatively more KLK4 present in the maturation stage of *Nckx4* ^*−/−*^ enamel matrix as compared to that in the *Nckx4* ^*+/+*^ enamel (see Figure 5E). A quantitative analysis on the density of WB immunoreactive bands confirmed that NCKX4 deletion did not affect MMP20 abundance in the secretory stage of enamel matrix, but resulted in a greater amount of KLK4 presence compared to the *Nckx4* ^*+/+*^ enamel matrix (see Figure 5F).

Through agar, fluorogenic quench peptide immobilized on the freshly microdissected maturation stage incisor segments was employed to visualize relative *in situ* KLK4 activity in the enamel matrix. The blue fluorescence signals from the hydrolyzed substrates (outlined by pink dotted line under the dentin-enamel junctions in Figure 5G), proportionally correspond to the KLK4 activity in the enamel matrix. The results demonstrated that KLK4 activity was decreased in *Nckx4* ^*−/−*^ enamel matrix, thus unable to completely process matrix proteins for enamel ECM modeling (see Figure 5H).

### KLK4 activity is independent of calcium concentration but is suppressed by increased sodium concentration

**FIGURE 6:**
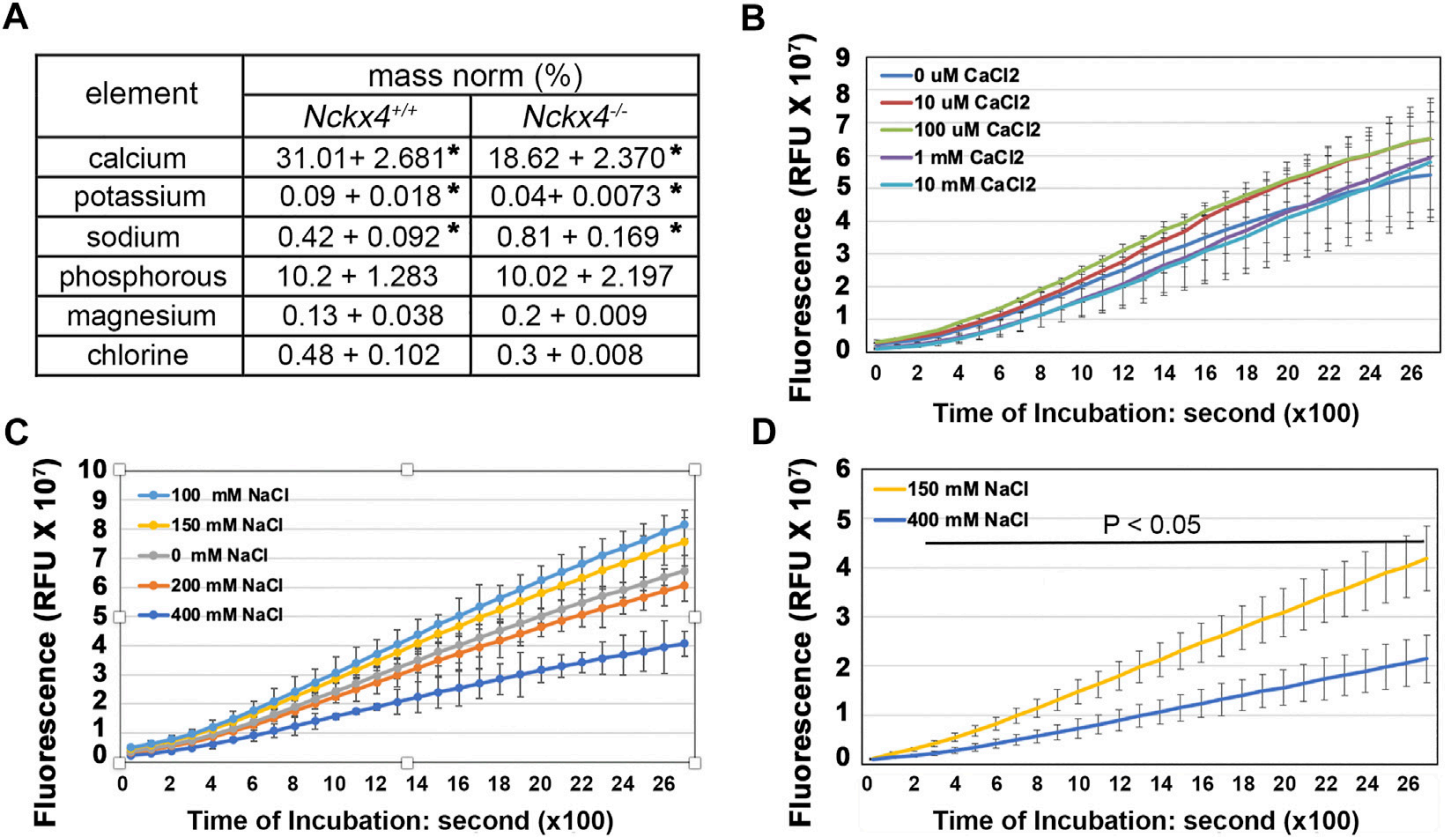
Determination of the effect of [Ca^2+^] and [Na^+^] on KLK4 activity. **(A)** An EDX analysis of the elements in the incisor centrolabial enamel underlying the mesial angle of the first molar showed reduced calcium and increased sodium in the *Nckx4* ^*−/−*^ matrix compared to that in the *Nckx4* ^*+/+*^ matrix. *n* = 5, *p < 0.05, determined by two-tailed unpaired Student’s *t*-test. **(B)** Recombinant KLK4’s ability to hydrolyze BOC-Val-Pro-Arg-AMC peptide was not affected by various [Ca^2+^]. **(C)** KLK4’s ability to hydrolyze BOC-Val-Pro-Arg-AMC peptide was maximal in 100 and 150 mM NaCl and reduced in 200 mM NaCl. The reduction of KLK4 activity in 400 mM NaCl was significant compared to the rest of the conditions after 800s incubation (*p* < 0.05). There was no significant difference in KLK4 activity in 100 mM NaCl compared to 150 mM NaCl. *n* = 4. **(D)** The maturation stage of *Nckx4* ^*+/+*^ incisor segments was more effective in hydrolyzing BOC-Val-Pro-Arg-AMC peptide in 150 mM NaCl than in 400 mM NaCl. The difference in KLK4 activity at any time point after 200-s incubation was significant (*p* < 0.05), *n* = 4. The significance in these enzymatic analyses was determined by two-way ANOVA analysis followed by Tukey Post Hoc Tests using SPSS Statistics package 19.

As NCKX4 is a known ion transport, we first evaluated to what degree that *Nckx4* deletion affected the ion composition in enamel matrix. EDX analysis, used to measure the elemental composition in incisor centrolabial enamel underlying the mesial angle of the first molars, showed reduced relative amounts of calcium and potassium by about 50%, while sodium increased about 50% in the *Nckx4* ^*−/−*^ enamel matrix as compared to that in the *Nckx4* ^*+/+*^ enamel (see Figure 6A).

The EDX data led us to measure the KLK4 peptidase activity in the presence of various concentrations of calcium and sodium. Its results showed that while KLK4 activity was independent of calcium concentration (see Figure 6B) but it was significantly decreased in the presence of high (200 and 400 mM) NaCl, both *in vitro* (see Figure 6C) and *in situ*, where shows the suppressed KLK4 activity in the maturation stage of *Nckx4* ^*+/+*^ enamel matrix (see Figure 6D). KLK4 activity was maximized when NaCl concentration was between 100 and 150 mM. This data implies that the transitorily increased sodium concentration in confined enamel matrix caused by defective NCKX4 could adversely affect KLK4 activity.

### KLK4 activity is suppressed by increased pH

**FIGURE 7:**
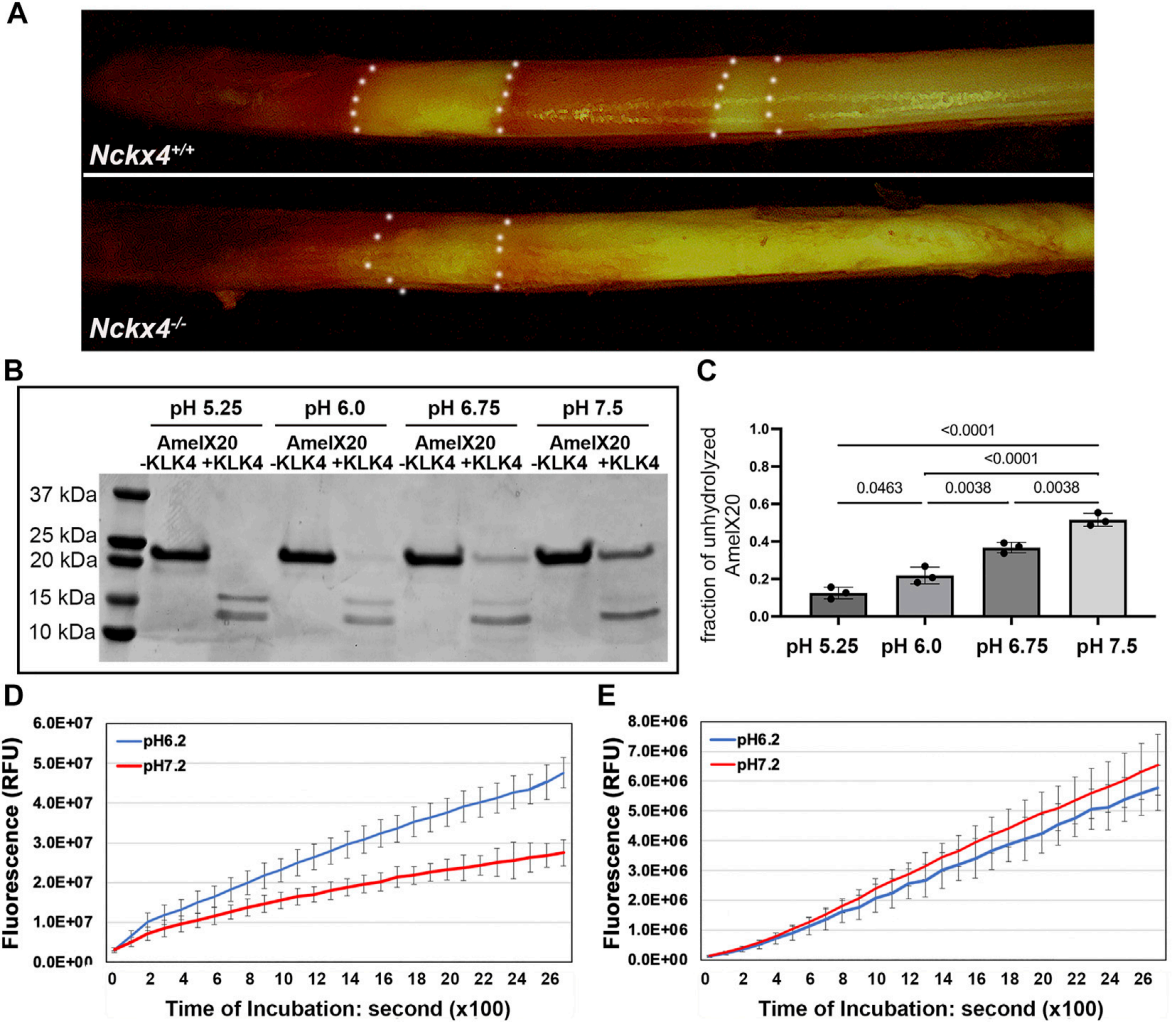
Determination of the effect of pH on KLK4 activity. **(A)** Methyl red staining revealed the cyclically alternating acidic pH bands (red) and neutral pH bands (yellow) presented on the P7W *Nckx4* ^*+/+*^ mouse incisor enamel but largely absent from the P7W *Nckx4* ^*−/−*^ mouse enamel. The boundary between acidic pH band and neutral pH band is outlined by the white dotted lines. **(B)** SDS-PAGE analysis of recombinant amelogenin 20 protein incubated overnight with or without recombinant KLK4 in buffer with various pH. **(C)** The protein band density analysis showed that the fraction of unhydrolyzed amelogenin 20 was significantly greater at pH 6.75 and pH 7.5, as compared to that in pH 5.25 and pH 6.0. Condition with pH 5.25 resulted in the least unhydrolyzed amelogenin 20, achieving the most effective digestion. Vice versa, condition with pH 7.5 gave rise to the lowest hydrolysis efficiency. The significance (*p*-value in the chart) was determined by one-way ANOVA analysis followed by Tukey Post Hoc Tests using SPSS Statistics package 19. **(D)** The maturation stage of *Nckx4* ^*+/+*^ incisor enamel hydrolyzed more peptides, releasing more fluorescence at pH 6.2 than at pH 7.2 at any time point after 200s incubation, *p* < 0.05, *n* = 4. The difference was determined by a two-tailed Student’s *t*-test. **(E)** There was no difference in the hydrolysis of BOC-Val-Pro-Arg-AMC peptides between pH 6.2 and pH 7.2 by the maturation stage of incisor enamel matrix when KLK4 was deleted.

We next asked if *Nckx4* deletion would affect enamel matrix acidification as a consequence of the reduced calcium phosphate formation? A pH indicator stain of freshly microdissected mandibular incisors showed that on the surface of *Nckx4* ^*+/+*^ enamel, acidic pH bands (red) and neutral pH bands cyclically alternated throughout enamel development (see Figure 7A, top panel). However, in the *Nckx4* ^*−/−*^ incisor enamel, the pH modulations failed to recur and the ECM remained neutral (see Figure 7A, lower panel).

To determine if this change in pH fluctuations affected KLK4 activity, we used an *in vitro* assay, in which the effect of pH on the ability of KLK4 to hydrolyze recombinant amelogenin 20 was measured. We used amelogenin 20 as the substrate since it is the major amelogenin species present in the early maturation stage of enamel matrix (Ryu et al., 1999). When the hydrolysis was performed in pH 5.25 or pH 6.0, there was less unhydrolyzed amelogenin 20 present on SDS-PAGE gel as compared to that in pH 6.75 or pH7.5 (see Figure 7B). The density analyses on the ratio of unhydrolyzed amelogenin20 vs the input protein confirmed that more amelogenin 20 was hydrolyzed in the acidic pH (see Figure 7C).

Furthermore, the ability of maturation stage *Nckx4* ^*+/+*^ incisor enamel matrix to hydrolyze fluorogenic peptide BOC-Val-Pro-Arg-AMC was also more effective in pH 6.2 than in pH 7.2 (see Figure 7D). These are the average pH found in enamel matrix under ruffle-ended ameloblasts and smooth-ended ameloblasts respectively. When there was no KLK4 present, the overall enzymatic activity of *Klk4* ^*−/−*^ mouse enamel matrix to hydrolyze BOC-Val-Pro-Arg-AMC peptide reduced to 11% of that in wild-type controls (see Figures 7D, E), indicating that KLK4 is responsible for about 89% of enzymatic activity of wild-type maturation stage of enamel matrix. There was no difference in the enzymatic activity of *Klk4* ^*−/−*^ enamel matrix in pH 6.2 and pH7.2 in term of hydrolyzing BOC-Val-Pro-Arg-AMC peptide, suggesting that other peptidases in *Klk4* ^*−/−*^ maturation stage of enamel were independent of pH.

## Discussion

Enamel is an extracellular matrix (ECM) that undergoes dynamic modeling in its matrix composition and microstructure, progressing from a protein-rich matrix to a highly mineralized matrix. Clinical genetics studies have shown that mutations in the human NCKX4 gene can cause amelogenesis imperfecta, a disorder resulting from enamel malformation (Parry et al., 2013; Seymen et al., 2014; Herzog et al., 2015; Jalloul et al., 2016). Through analyzing the enamel matrix ion composition and matrix pH on mouse incisors, where the trajectory of enamel matrix modeling is fully present, and *in situ* and *in vitro* proteinase KLK4 activity, we identified an additional function of NCKX4 in regulating enamel matrix protein hydrolysis and enamel composition modeling. Our results showed that the activities of both recombinant and mouse enamel matrix native KLK4 were [Na^+^]- and pH-dependent. By optimizing sodium concentration and pH in the encompassed enamel matrix compartment, NCKX4 promotes KLK4-mediated enamel matrix protein hydrolysis. Breaking down the enamel matrix proteins is necessary for ECM protein removal to give rise to a complete biomineralized enamel tissue.

In agreement with previous studies (Hu et al., 2012; Wang et al., 2015; Bronckers et al., 2017), we confirmed that NCKX4 was primarily present in maturation ameloblasts (MAB) during normal enamel development. In addition, we illustrated the dynamic expression pattern of *Nckx4* transcript from the transition stage of ameloblasts, to early maturation stage of ameloblasts, then to middle and maturation ameloblasts. Our immunostaining analyses on mouse incisor sagittal sections also localized NCKX4 predominately at the apical surface of ruffle-ended maturation ameloblasts (REMAs). This data further supports the significance of REMAs in transporting abundant minerals into the enamel matrix, facilitating the transformation of enamel from protein matrix to highly mineralized tissue (Smith, 1998; Katsura et al., 2014; Wang et al., 2015).

Our finding of a reduction of calcium (by approximately 50%) in the *Nckx4* ^*−/−*^ mouse enamel compared to wild-type controls supports the role of NCKX4 as a major player in transporting calcium into the enamel matrix. We found that the expression levels of *Pmca* and *Ncxs*, molecules at the apical surface of ameloblasts that are also known to extrude calcium to enamel matrix (Nurbaeva et al., 2017; Robertson et al., 2017), were unchanged in *Nckx4* ^*−/−*^ maturation ameloblasts compared to *Nckx4* ^*+/+*^ ameloblasts by semi-quantitative PCR (data not shown). The data implies that these molecules were able to transport some amount of calcium but could not fully compensate for the loss of NCKX4.

In contrast to the wild-type mouse enamel, where ECM proteins are progressively processed and diminished, matrix proteins failed to be removed from the maturation stage of *Nckx4* ^*−/−*^ mouse enamel. Our SDS-PAGE and western blot analyses showed a retention of amelogenin 20 (the amelogenin with C-terminus removed by MMP20) in the *Nckx4* ^*−/−*^ enamel matrix. This result suggests that in addition to providing calcium, NCKX4 contributes to the hydrolysis of enamel matrix proteins at the maturation stage of amelogenesis. In principle, enamel matrix proteins of the secretory stage are primarily hydrolyzed by MMP20 (Bartlett et al., 2011); in the proceeding maturation stage, KLK4 is the primary enzyme to hydrolyze the remaining amelogenin and other EMPs (Ryu et al., 2002; Lu et al., 2008; Yamakoshi et al., 2011; Yamakoshi et al., 2013). Our quantitative enzyme activity analysis showed that KLK4 takes up about 90% of proteinase responsibility in the maturation stage of the enamel matrix. Consistent with our observation in which ameloblast morphology and underlying matrix proteins were unaffected in the secretory stage, we did not detect any significant difference in the expression of ECMs and MMP20 between *Nckx4* ^*+/+*^ and *Nckx4* ^*−/−*^ secretory ameloblasts either (data not shown). Therefore, NCKX4 ablation does not affect the production and hydrolysis of enamel matrix proteins in the protein phase of enamel matrix modeling. Surprisingly, we found increased *Klk4* mRNA in the *Nckx4* ^*−/−*^ maturation ameloblasts and KLK4 protein in the *Nckx4* ^*−/−*^ matrix. However, *in situ* KLK4 activity of the *Nckx4* ^*−/−*^ enamel matrix was reduced.

Intuitively, we first focused on investigating whether the reduced calcium in the *Nckx4* ^*−/−*^ enamel is responsible for the reduced KLK4 activity. Using fluorescence released from a quenched peptide (a FRET peptide) as a readout of KLK4 activity, we demonstrated that KLK4 activity was calcium-independent. Considering calcium is such an abundant element in the enamel compartment encompassed by the dentin matrix and epithelial barrier, we believe that calcium-independent propensity would be advantageous to the KLK4’s enamel tissue-specific function. It would be very challenging to elaborately control KLK4’s activity if its activity is sensitive to calcium concentration in the confined enamel microenvironment. Overpowered KLK4 could impair the anatomy of the dentin and ameloblast layer. Thus, KLK4’s calcium-independent property is critical for dentin and enamel formation.

Although KLK4 activity is calcium-independent, calcium may still indirectly affect KLK4 activity in the *Nckx4* ^*−/−*^ enamel matrix through MMP20. MMP20 is a well-known calcium-dependent metalloproteinase, which can activate KLK4 *in vitro* (Wang et al., 2006; Hadler-Olsen et al., 2011; Yamakoshi et al., 2013). Thus, the efficiency of MMP20 remaining in the early maturation stage of the enamel matrix may be adversely affected by the reduced calcium, subsequently decreasing the activity of KLK4 in the maturation stage of *Nckx4* ^*−/−*^ enamel matrix. Reduced MMP20 activity in the early maturation stage of the enamel matrix may also drive the compensatory upregulation of *Klk4* mRNA in the *Nckx4* ^*−/−*^ maturation ameloblasts.

We next assessed the effect of sodium on KLK4 activity since we also found about a 50% increase in sodium in the maturation stage of the *Nckx4* ^*−/−*^ enamel matrix. Our analysis demonstrated that KLK4 activity was greater when sodium concentration ranged from 100 mM to 150 mM than sodium at 200 mM and 400 mM. The activity of KLK4 started to decline in 200 mM NaCl and dropped more significantly in 400 mM NaCl. The sodium concentration in the secretory stage porcine enamel fluid is 140 mM (Aoba and Moreno, 1987). Given that the enamel matrix is a microenvironment confined by the dentin matrix and tightly junctional epithelial barrier, sodium concentration may likely go beyond the normal range transitorily when the major transport NCKX4 failed to remove sodium from the enamel matrix. The significance of sodium concentration in the enamel matrix protein hydrolysis is also evidenced by the previous NMR studies, which demonstrate that a high salt concentration salt promotes amelogenin nanosphere self-assembly (Buchko et al., 2008; Shaw et al., 2020), forming a complex that is less accessible to proteinases.

Removing as much sodium as possible during normal development is necessary to make adequate space for calcium and phosphate (Bronckers et al., 2015) and balance the microenvironment’s osmotic pressure. Subameloblastic cysts were often found in the maturation phase of the *Nckx4* ^*−/−*^ enamel development (see Figure 5C), which is the additional evidence indicating the increased osmotic pressure possibly resulting from above physiological levels of sodium concentration. This increased sodium concentration may partially impair KLK4 activity. The high osmotic pressure could also subsequently affect ameloblast morphology and functions.

Metal ions play important roles in many enzyme-catalyzed reactions, such as magnesium to CCA-adding enzymes (Hou et al., 2005), zinc and calcium to matrix metalloproteinases (Tezvergil-Mutluay et al., 2010), and sodium to NADH dehydrogenase (Tuz et al., 2015). Metal ions can regulate an enzyme’s kinetics by influencing the enzyme’s conformation, redox states, and enzyme-substrate binding ability (Badarau and Page, 2006; Page and Di Cera, 2006; Page et al., 2006; Tuz et al., 2015). Monovalent cations Na^+^ and K^+^ can also act as non-specific buffering agents, solute exchange mediators, and enzyme stabilization agents. Levinsky *et al.* have shown that the maximum activity of urinary kallikrein is achieved in 100 mM sodium (Lieberthal et al., 1982), a similar condition that optimizes KLK4 activity in the enamel. The regulatory mechanism underlying how sodium affects the kinetic property of KLK4 remains to be determined by future studies.

During the maturation stage, calcium combines with phosphate to form hydroxyapatite crystals and produce protons, resulting in multiple acidified zones separated by the neutral pH zone on the enamel surface, a unique phenomenon called as pH cycling (Takano et al., 1988; Takano et al., 1989; Smith, 1998). As expected, with the deletion of NCKX4 and reduced ruffle-ended ameloblast-mediated calcium extrusion activity, the maturation stage enamel matrix was less acidified and largely remained neutral. Previous studies have reported that overall, enamel matrix proteins are processed more rapidly in the zones with lower pH than in the zones with neutral pH (Smith et al., 1996; Lu et al., 2008). Our *in vitro* studies demonstrated that recombinant KLK4 enzyme hydrolyzed the recombinant amelogenin 20 more effective at the lower pH up to 5.25 than at pH 7.0. Amelogenin 20, as a MMP20 hydrolyzed amelogenin product, is the primary amelogenin species present in the maturation stage enamel. These data help us further appreciate the significance of enamel matrix pH cycling in ECM modeling. We plan to investigate how pH affects KLK4 hydrolysis activity in future studies. Full-length amelogenin’s structure is sensitive to pH, with increasing self-assembling propensity as the microenvironment pH increases (Delak et al., 2009; Zhang et al., 2011; Shaw et al., 2020). We hypothesize that acidic pH promotes amelogenins to form less complicated assemblies, which may be more accessible to KLK4 docking.

Our analyses also conclude that KLK4 accounts for about 89% of protein hydrolysis activity presented by the normal maturation stage enamel matrix. Other peptidases in the enamel matrix are responsible for the rest of 11% of hydrolysis activity. Furthermore, the remaining non-KLK4-associated proteinase activity is not pH-dependent. Being more effective in acidic pH may contribute to KLK4’s proteinase specificity in the enamel matrix. KLK4 is a soluble and vigorous proteinase, therefore, suppressing its activity by neutral pH may be a mechanism for preventing dentin matrix proteins and ameloblast cell surface proteins from undesired hydrolysis by KLK4.

**FIGURE 8:**
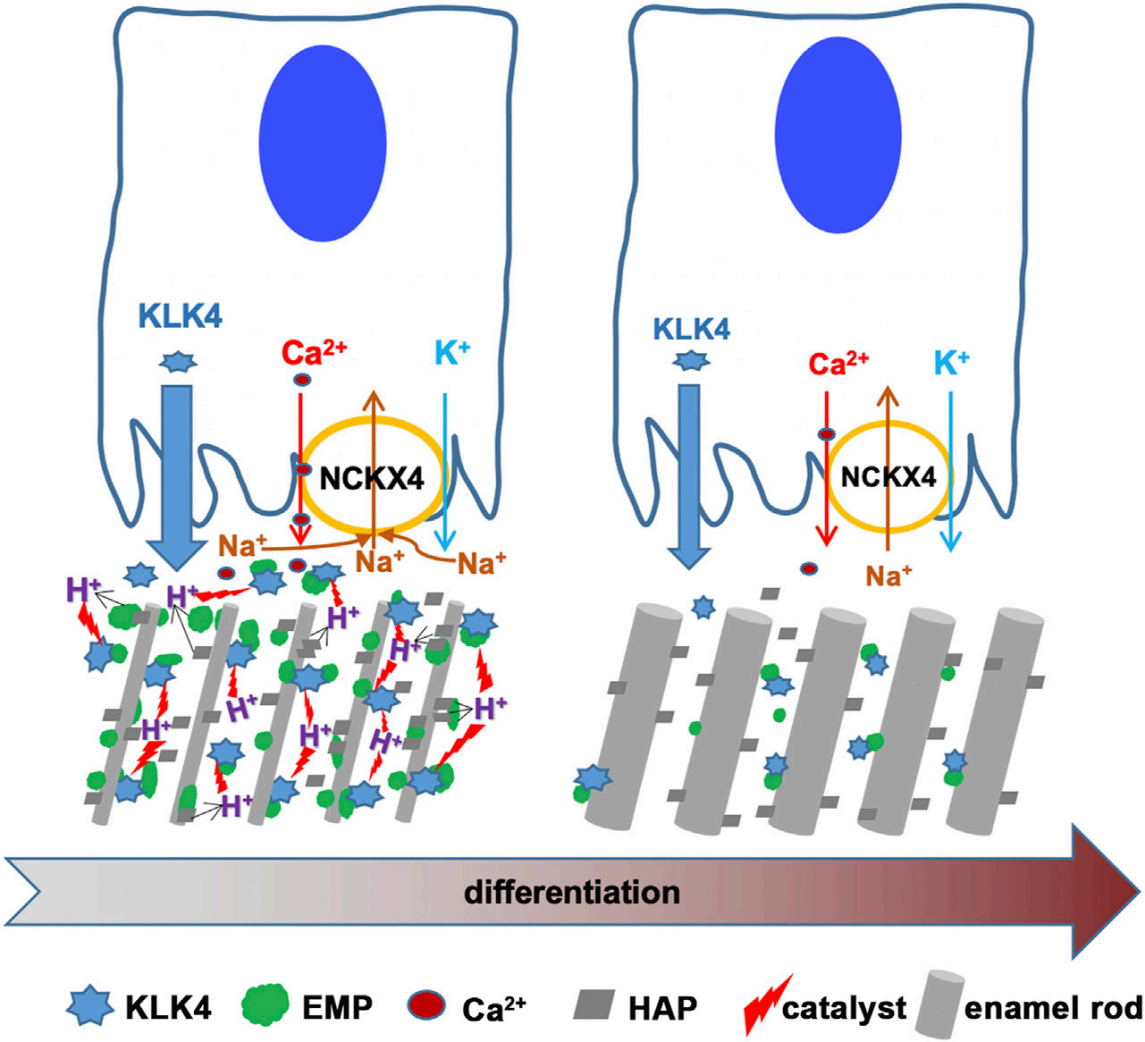
This scheme is to depict the functions of NCKX4 in calcium transport and matrix protein removal during enamel ECM modeling. KLK4 and NCKX4 are significantly upregulated in the early and mid-maturation stages of ameloblast maturation. However, the expression of both proteins declines as maturation continues. Immunostaining analysis of the full course of progressively differentiating ameloblasts localizes NCKX4 primarily at the apical surface of ruffle-ended maturation ameloblast (RAB). The molecular mechanisms of how NCKX4 interacts with KLK4 to regulate the enamel matrix modeling are illustrated here. NCKX4 transports calcium, which interacts with phosphate to produce Ca_10_(PO4)_6_(OH)_2_ hydroxyapatite (HAP). HAP incorporates preexisting slender enamel rods constructed by secretory ameloblasts to grow enamel rods in width. Formation of each unit of HAP releases (10Ca^2+^ +6HPO4^2-^ + 2H_2_O → Ca_10_(PO4)_6_(OH)_2_ + 8H^+^) eight protons into enamel ECM. These protons acidify ECM, which accelerates KLK4 to hydrolyze matrix proteins, facilitating them to be removed by ameloblasts to make space for progressive mineral incorporation. While transporting calcium into ECM, NCKX4 removes sodium from the enamel matrix. By coordinating the pH and sodium concentration in enamel ECM, a novel function of NCKX4 is to regulate the activity of KLK4 to model the enamel from a proteinaceous matrix into the most mineralized tissue in our body.

Taken together, by delivering calcium into the enamel, which facilitates hydroxyapatite formation, and moving off alkali ion sodium from enamel matrix, NCKX4 coordinately shifts pH and [Na^+^] in the enamel matrix, which accelerates KLK4 to hydrolyze and remove ECM proteins. This function of NCKX4 permits the progressive enamel matrix modeling and mineralization (see Figure 8).

KLK4-hydrolyzed enamel matrix proteins can be removed from enamel matrix by ameloblasts through endocytosis to make space for minerals. Therefore, KLK4 activity reduction caused by NCKX4 deletion may secondarily affect the ameloblast’s endocytosis function*.* Thus, impaired endocytosis could also be partially responsible for the defective matrix protein retention in the *Nckx4* ^*−/−*^ enamel. In addition, we found that the deletion of NCKX4 disrupted ameloblast polarity, as indicated by the disperse distribution of PKC alpha (Figure 3), an epithelial polarization marker (Smith et al., 2007). The disrupted polarity may affect the formation of membrane infoldings, required by endosome formation at the interface between ECM and ameloblasts (Salama et al., 1987).

Identifying the factors that regulate KLK4 activity is important for enamel biology and enamel tissue regeneration and may also shed light on cancer biology. KLK4 has been identified as an indicator for breast, prostate, and ovarian cancer progression (Obiezu et al., 2001; Papachristopoulou et al., 2009; Lose et al., 2012; Wang et al., 2018). Regional hypoxia can induce tumor microenvironment acidification during tumor progression, which promotes the cancer cells to traverse the ECM barrier and invade local circulation for metastasis (Webb et al., 2011; Doe et al., 2012; Huang et al., 2016; Asgharzadeh et al., 2017). Shall elevated sodium concentration or/and microenvironment pH be able to inhibit KLK4 ability to clean the ECM barrier and constrain tumor cell migration? Understanding the regulation of KLK4 catalytic mechanisms may inspire us to devise potential clinical strategies to intervene in the progression of cancer as well.

## Experimental procedures

### Animals

NCKX4 loss-of-function mouse colony (*Nckx4* ^*−/−*^) with deletion of exon 6 and 7 (Li and Lytton, 2014) is a gift from Dr. Jonathan Lytton. KLK4 loss-of-function mouse colony (*Klk4* ^*−/−*^) was purchased from The Jackson Laboratory. All animals were maintained in the UCSF animal care facility, which is a barrier facility, accredited by Association for Assessment and Accreditation of Laboratory Animal Care (AAALAC). All experimental procedures associated with these mouse models were approved by the Institutional Animal Care and Use Committee (IACUC) under the protocol AN183449-01.

### Histology and immunostaining assessment

Mice at postnatal seven-week-old (P7W) were collected by following standard IACUC protocols. Briefly, mice were anesthetized with 240 mg/kg tribromoethanol (Sigma-Aldrich), and perfuse-fixed with PBS and 4% paraformaldehyde (PFA). Hemimandibles were dissected and post-fixed with 4% PFA for 24 h at 4°C, then followed by decalcification in 8% EDTA at 4°C for 3 weeks with EDTA change every other day. The hemimandibles were then processed, embedded with wax and sectioned along their sagittal planes. Sections were stained with Hematoxylin & Eosin (H&E) for morphology assessment.

For immunostaining, the sagittal sections were boiled in 10 mM citrate buffer (pH 6.0) for 20 min to retrieve antigens. Next, the sections were incubated with GeneTex Universal Protein Blocking reagent for 1 h, followed by incubation with mouse anti-NCKX4 antibody (UC Davis/NIH NeuroMab Facility) or rabbit anti-amelogenin antibody for overnight at 4°C. Sections were then incubated with Alexa 594 fluorescein-conjugated anti-mouse IgG or FITC-conjugated anti-rabbit IgG (Life Technologies) for 1 h at RT, followed by counterstaining with 1 μg/mL Hoechst for 5 min (Life Technologies). The slides were imaged using a high-speed Leica TCS SP5 spectrum confocal microscope. To evaluate ameloblast polarity, after blocking with Universal Protein Blocking reagent, the sections were incubated with rabbit anti-PKC (protein kinase C) alpha antibody (Abcam) for overnight at 4°C. After washing, the sections were then incubated with HRP-conjugated goat anti-rabbit antibody for 1 h at RT, at last incubated with HRP substrate (Vector®) to reveal the positive immunoreactive signals.

### *In situ* RNA hybridization

RNAscope *in situ* hybridization 2.5 HD Red Detection Kit and mouse *Nckx4* and *Klk4* probes (Advanced Cell Diagnostics, ACDbio.) were used by following the manufacturer’s instructions. Briefly, sagittal sections of mouse hemimandibles at 5 µm of thickness were boiled in the target retrieval buffer for 15 min and then pre-treated with protease plus solution at 40°C for 15 min. The sections were next incubated with probes for 2 h. After incubating with a serial of amplification reagents, the sections were incubated with Fast Red complex to develop the red precipitates in which the intensity is proportional to the mRNA expression levels of target genes. The nuclei were lightly counterstained by hematoxylin.

### Macro-morphology photography and X-ray micro-tomography

P7W mouse hemimandibles were air-dried prior to imaging with a Leica M165C digital stereo microscope and a LAS V4.2 software. Hemimandibles were then subjected to X-ray micro-tomography scanning using a Micro XCT-200 system (Xradia). All scans were done at an operating voltage of 90 KVp and 66 μA of current, at an optical magnification ×2. A binning of 2 was used for 3D image reconstruction. Virtual sections were converted to bmp images using the Xradia TXM3DViewer 1.1.6. software. Mineral densities were determined following a detailed calibration protocol of the micro-XCT described previously (Djomehri et al., 2015). Ten orthogonal sections from each mouse (*n* = 3) to the long-axis of hemimandible at the plane perpendicularly across either the second buccal cusps of the first molar (P1 in Figures 2E, F) or the onset of mesial angle of the first molar (P2 in Figures 2E, F) were collected for mineral density analysis. The mineral density of calcified tissues from *Nckx4* ^*+/+*^ and *Nckx4* ^*−/−*^ mice was compared using unpaired two-tailed Student’s *t*-test.

### Assessment of expression levels of genes of interest by semi-quantitative PCR

Pups at postnatal day 5, 7, 8, 9, 11, and 13 (P5, P7,P8, P9, P11, and P13) were euthanized with carbon dioxide asphyxiation followed by cervical dislocation, mandibles were dissected and first molars from five mandibles were extracted and pooled as one sample. Enamel epithelium overlying these first molars is at the secretory, transition and maturation stage of development respectively (Ryu et al., 2002). The total RNA of these cells was purified using Qiagen RNeasy Micro Kit and mRNAs were reverse-transcribed into cDNA libraries using SuperScriptTM III First-Strand Synthesis System (Life Technologies). Semi-quantitative PCR was performed to quantify the relative expression levels of target genes. The sequences of primers used to amplify mouse endogenous *Gapdh* and target genes were listed as follows: *Gapdh* sense- 5′-TGG​CCT​TCC​GTG​TTC​CTA​C-3′, antisense- 5′-GAG​TTG​CTG​TTG​AAG​TCG​CA-3′; *Nckx4* sense- 5′- CAC​GGA​GAT​GTC​GGT​GTA​GGA-3′, antisense- 5′- CAC​CAC​CTG​TCC​AGC​AAA​GAG-3′; amelogenin sense- 5′-GGG​ACC​TGG​ATT​TTG​TTT​GCC-3′, antisense- 5′-TTC​AAA​GGG​GTA​AGC​ACC​TCA;-3′; enamelin sense- 5′-GCT​TTG​GCT​CCA​ATT​CAA​A-3′, antisense- 5′-AGG​ACT​TTC​AGT​GGG​TGT-3′, ameloblastin sense- 5′-CTG​TTA​CCA​AAG​GCC​CTG​AA-3′, antisense- 5′-GCC​ATT​TGT​GAA​AGG​AGA​GC-3′; *Mmp20* sense- 5′-TCC​AAG​CAT​TAT​ACG​GAC​CCC-3′, antisense- 5′-GTC​ACT​GCA​TCA​AAG​GAC​GAG-3′; *Klk4* sense- 5′-CGG​GAG​TCT​TGG​TGC​ATC​C -3′, antisense- 5′- CTT​GGG​AGC​CTT​TCA​GGT​TAT​G -3′. To determine the relative expression levels of these target genes, the comparative threshold cycle method was used as previously published (Ryu et al., 1999).

### Scanning electron microscopy (SEM)

Three sets of hemimandibles from either P7W *Nckx4* ^*+/+*^ or *Nckx4* ^*−/−*^ mice were embedded in epoxy, and polished in an orientation crossing to the long-axis of incisor using a series of SiC paper and diamond polishing suspension. Subsequently specimens were etched with 10% HCl for 30 s. Specimens were sputter-coated with Au-Pd, and the thickness of incisal enamel matrix and enamel rods were imaged at 20 kV using a Scanning Electron Microscope, Quanta 3D FEG (FEI) as described previously (Habelitz, 2015).

### SDS-PAGE protein analysis

Hemimandibles were dissected, alveolar bone at the lingual site of hemimandibles was carefully removed to expose incisors. Incisors were fragmented using molars as reference line as previous published (Hu et al., 2007). The incisor segments under the second and third molars were used to extract secretory stage enamel matrix. The incisor segments from the bottom of the first molar to the gingival margin were used to extract the maturation stage matrix. The stage-specific enamel matrix proteins were extracted as previously described (Yamakoshi et al., 2011). The incisor segments from 6 mice were pooled and incubated in 1 mL of 0.17 N HCl/0.95% formic acid for 2 h at 4°C with constant rotation. After the undissolved material was removed by centrifugation at 3500g in 4°C, the protein supernatant was subjected to buffer exchange with 0.01% formic acid using a centrifugal 3K-filter unit (EMD Millipore). The proteins retained by the filter were eluted with 250 μL of 0.01% formic acid and used for subsequent sodium dodecyl sulfate polyacrylamide gel electrophoresis (SDS-PAGE), Coomassie Brilliant Blue staining, and western blot immunoblotting.

### Western blot analyses

Equal volumes of total enamel matrix proteins from six mice at either secretory stage or maturation stage were loaded to be separated on a 15% SDS-PAGE gel. Proteins on the SDS-PAGE were transferred to a PVDF membrane. After blocking, membrane was then incubated with rabbit anti-amelogenin IgG, rabbit anti-MMP20 IgG (Millipore-Sigma), or rabbit anti-KLK4 IgG (Abcam) for overnight at 4°C. Following washing, PVDF was secondly incubated with IRDye 680RD conjugated anti-rabbit IgG (Li-Cor) for 1 h at room temperature. The membranes were thoroughly washed and then scanned using an Odyssey Imaging System.

### KLK4 activity assessment

The activity of KLK4 to hydrolyze a peptide substrate or recombinant protein amelogenin 20 (amelogenin without C-terminus) was analyzed in various buffer. Briefly, recombinant KLK4 (R&D Systems) was activated by thermolysin (R&D Systems) by following the manufacturer’s instruction and previous publications (Yoon et al., 2007; Tye et al., 2011). Active KLK4 was then incubated with a fluorogenic quench peptide substrate BOC-Val-Pro-Arg-AMC (R&D Systems) in the 50 mM Tris-HCl supplemented with various concentrations of CaCl_2_ or NaCl. To test the effect of pH, we used buffer supplemented with 50 mM Tris-HCl, 150 mM NaCl and 10 mM CaCl_2_. Once the Arg-AMC amide bond in the peptide is hydrolyzed by KLK4, highly fluorescent AMC is released. The intensity of fluorescence proportionally indicates the activity of KLK4. One hundred microliter reaction was loaded to each well of a 96-well black plate. Each condition was quadruplicate. The plate was read at excitation and emission wavelengths of 380 nm and 460 nm respectively. A kinetic mode was set to read each well every 100 s for a total of 2,700 s interval.

To synthesize amelogenin 20, also named as rH146 in previous publication (Bai et al., 2020), a site-directed mutagenesis kit (Takara Bio.) was employed to delete C-terminus 28bp DNA from human wild-type amelogenin cDNA (rH174). After the site-specific deletion was confirmed by DNA sequencing, amelogenin 20 was expressed in T7 Express Competent *Escherichia coli* (New England Biolabs) and purified using an acid/heat treatment method described previously (Svensson Bonde and Bulow, 2012). Amelogenin 20 recombinant protein was incubated with or without active KLK4 (at a concentration ratio of 100:1) in 50 mM Tris-HCl, 150 mM NaCl and 10 mM CaCl_2_ at various pH for overnight at 37°C with rotation. Then protein was loaded to a 15% SDS-PAGE. The gel was scanned and protein band density was measured using NIH ImageJ version 2.0.0. The percentage of undigested protein normalized to the input amelogenin 20 was calculated. An average of percentage from triplicate analyses was calculated and the significant difference among the different conditions was determined by one-way ANOVA analysis using SPSS Statistics package 19 followed by Tukey Post Hoc Tests.

To analyze the activity of native KLK4 possessed in the maturation phase of enamel matrix, the incisor segments overlying the bottom of the first molar to the gingival margin (maturations stage) were microdissected and incubated with fluorogenic peptide BOC-Val-Pro-Arg-AMC. The fluorescence was recorded every 100 s for a total of 2,700 s interval by a plate reader. To assess KLK4 enamel *in vivo* activity, the freshly microdissected incisor segments at the maturation stage as described above were quickly dipped into 1% agar in 50 mM Tris-HCl (pH 7.5), 150 mM NaCl and 10 mM CaCl_2_ supplemented with fluorogenic peptide BOC-Val-Pro-Arg-AMC. One microliter peptide was supplemented to each 100-μL of 1% agar. Next, the incisor segments embedded by agar were incubated in a black Eppendorf tube for 30 min at 37°C. After incubation was complete, the incisal segments were photographed immediately using a Leica fluorescence microdissection microscope.

### Enamel matrix element measurement

Hemimandibles from three 7-week old mice were embedded in epoxy, and polished in a cross orientation using a series of SiC paper and diamond polishing suspension starting from incisor tip and stopping when mesial angle of the first molar was exposed. For each hemimandible, six randomly areas (as illustrated in Supplementary Figure S1) were subjected to an energy dispersive X-ray spectroscopy (Quantax EDS, Bruker Nano Inc.) to collect elemental composition of Ca, P, Na *et al* at 15 keV using a variable pressure chamber. Back scattered electron (BSE) images were also collected using a field emission scanning electron microscope (Sigma VP500 field emission SEM, Carl Zeiss Microscopy). The elemental composition collected from three *Nckx4* ^*+/+*^ or *Nckx4* ^*−/−*^ mice was compared and the significance difference between two mouse models was determined by unpaired two-tailed Student’s t-test.

### Mouse incisor enamel matrix pH staining

To prepare the staining solution, 100 mg Methyl Red (Sigma-Aldrich) was diluted in 45 mL of 95% methanol. Four *Nckx4* ^*+/+*^ or *Nckx4* ^*−/−*^ mice at postnatal 7 weeks old (P7W) were anesthetized with 240 mg/kg tribromoethanol (Sigma-Aldrich). Hemimandibles were dissected and the surrounding alveolar bone was removed to completely expose the mandibular incisors. The enamel organ epithelium overlying the labial enamel surface was gently wiped off with ice-cold Kimwipe. The incisors were immediately soaked in 0.2% Methyl Red/95% methanol for 45 s at room temperature, then air-dried for 5 min prior to documenting the stained incisors under a Leica dissecting microscope.

## Data Availability

The original contributions presented in the study are included in the article/Supplementary Material, further inquiries can be directed to the corresponding author.

## Supplementary material

SUPPLEMENTARY FIGURE S1 For each hemimandible from the *Nckx4* ^*+/+*^ or *Nckx4* ^*-/-*^ mouse, six randomly areas (as illustrated in those yellow boxes) were subjected to an energy dispersive X-ray spectroscopy to collect elemental composition.
