# Supplementary material for: Sodium/(calcium + potassium) exchanger NCKX4 optimizes KLK4 activity in the enamel matrix microenvironment to regulate ECM modeling
Source: Front Physiol. 2023 Feb 6;14:1116091. doi: 10.3389/fphys.2023.1116091 (PMC9939835; doi:10.3389/fphys.2023.1116091)
Supplement: Supplementary file 1 [file DataSheet1.docx]

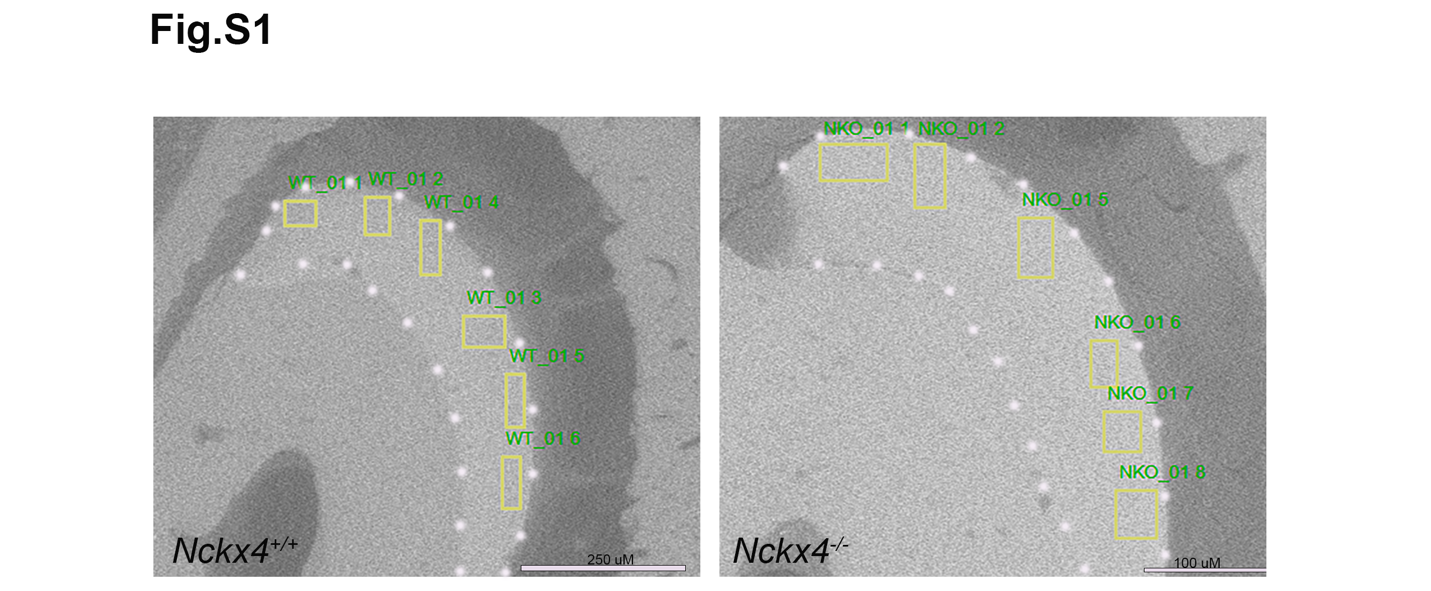


Fig.S1: For each hemimandible from the *Nckx4^+/+^* or *Nckx4^-/-^* mouse, six randomly areas (as illustrated in those yellow boxes) were subjected to an energy dispersive X-ray spectroscopy to collect elemental composition.
